# Association between glycated hemoglobin and functional outcomes in patients with intracranial large artery atherosclerotic disease-related acute ischemic stroke: identifying the magic number

**DOI:** 10.3389/fneur.2023.1249535

**Published:** 2023-09-27

**Authors:** Azra Zafar, Aishah Albakr, Rizwana Shahid, Fahd Alkhamis, Majed Alabdali, Danah Aljaafari, Saima Nazish, Foziah Jabbar Gossab AlShamrani, Erum Shariff, Mohammad Zeeshan, Abdulla AlSulaiman, Abdullah Saleh AlAmri, Anas Salman Aldehailan, Hosam Al-Jehani

**Affiliations:** ^1^Department of Neurology, College of Medicine, Imam Abdulrahman Bin Faisal University, Dammam, Saudi Arabia; ^2^Department of Medical Education, College of Medicine, Imam Abdulrahman Bin Faisal University, Dammam, Saudi Arabia; ^3^Department of Neurosurgery, Critical Care Medicine, and Interventional Radiology, Imam Abdulrahman Bin Faisal University, Dammam, Saudi Arabia

**Keywords:** glycated hemoglobin, HbA1c level, acute ischemic stroke, intracranial large artery atherosclerotic disease, functional outcomes, predictor

## Abstract

**Objective:**

This study aimed to investigate the effect of the glycated hemoglobin A1c (HbA1c) level on the functional outcome (FOC) in patients with intracranial large artery atherosclerotic disease (ICLAD)-related acute ischemic stroke (AIS).

**Methods:**

This retrospective study enrolled patients with ICLAD-related AIS who were admitted to King Fahd University Hospital between January 2017 and September 2021. Patients were divided into two groups based on the optimal cutoff HbA1c level determined using receiver operating characteristic curve analysis—those with HbA1c ≤6.9% and those with HbA1c >6.9%. Demographic and other clinical characteristics were compared between the two groups using chi-square tests. The association between HbA1c and 90-day FOC was assessed using the chi-square test and odds ratios (ORs). Multivariate analysis was performed to adjust for confounding factors.

**Results:**

A total of 140 patients were included in the analysis. A significant association was observed between the HbA1c level and FOC. Compared to patients with HbA1c ≤6.9%, patients with HbA1c >6.9% were more likely to have an unfavorable FOC [*p* = <0.001, OR = 2.05, 95% confidence interval (CI) = 1.33–3.14]. The association between HbA1c >6.9% and unfavorable FOC was sustained even after adjusting for confounding factors (*p* = 0.008) and atherosclerosis risk factors (*p* = 0.01). HbA1c >6.9% was also associated with higher ORs for in-hospital complications (*p* = 0.06, OR = 1.34, 95% CI = 1.02–1.77) and mortality (*p* = 0.07, OR = 1.42, 95% CI = 1.06–1.92) although these associations did not attain significant *p*-values.

**Conclusion:**

HbA1c >6.9% was significantly associated with unfavorable FOC in ICLAD-related AIS. However, further studies with larger sample sizes are required to verify whether HbA1c is an independent predictor of poor FOC. Nevertheless, targeting HbA1c <7% should be the goal of physicians when managing patients at high risk of ICLAD.

## 1. Introduction

Intracranial large artery atherosclerotic disease (ICLAD) is a subtype of the large artery atherosclerosis (LAA) etiology affecting the intracranial circulation, which is considered the most common cause of stroke worldwide (1). ICLAD accounts for 20–50% of cases of acute ischemic stroke (AIS) in Asian countries, including Saudi Arabia (SA) (1, 2). The relationship between glycated hemoglobin (HbA1c) levels and atherosclerotic cardiovascular diseases (CVDs), including cerebrovascular disease, is well established, and HbA1c ≥7% is associated with increased risks of CVD morbidity and mortality, including stroke (3). The HbA1c level, which is a marker of the average glycemic level over the past 2–3 months, is also an independent risk factor for atheromatous plaque development in vessels, irrespective of a diagnosis of diabetes mellitus (DM), and the risk escalates significantly with HbA1c Top of Form ≥7% (4). Moreover, HbA1c is not only an important factor for the development of atherosclerotic changes that lead to stroke but also affects its outcomes. The adverse effect of admission hyperglycemia and an elevated HbA1c level on stroke outcomes has been established in various studies, including those wherein intravenous thrombolysis and endovascular thrombectomy (EVT) were performed (5–8).

Researchers from China have investigated the effect of admission HbA1c on functional outcomes (FOC) in AIS (7–9). Gao et al. found HbA1c ≥6.7% to be associated with an unfavorable outcome in 793 patients with AIS due to small vessel occlusion (7), whereas Wang et al. identified HbA1c ≥6.5% as an independent risk factor for adverse outcomes among 408 patients with AIS (8). Moreover, Dong et al. analyzed 326 patients with acute anterior ischemic circulation stroke and reported that HbA1c >8% was significantly correlated with a poor FOC, which persisted after adjustment for confounding factors in patients with LAA stroke subtypes (*n* = 182) but not in those with non-LAA subtypes (9). Apart from these studies, some studies have reported HbA1c ≥7% to be significantly associated with poor FOC in AIS (10). However, the effect of HbA1c level on FOC in ICLAD-related AIS, which is the most prevalent subtype of stroke, has not been widely studied and is therefore not clear. Thus, the present study aimed to identify the HbA1c value predictive for unfavorable FOC in patients with ICLAD-related stroke to optimize the management of those at risk and to provide evidence regarding the HbA1c target value. Identifying the relationship between chronic hyperglycemia and FOC is important as the American Diabetes Association (ADA) recommends maintaining HbA1c at levels <7% to prevent CVD morbidity and mortality (11). The results of this study could have a significant impact on health practices in SA and reduce morbidity and disease-related burden.

## 2. Materials and methods

This retrospective study was conducted in the Department of Neurology of King Fahd Hospital of the University. The study enrolled patients admitted with AIS between January 2017 and September 2021. The study was approved by the Institutional Ethical Committee of Imam Abdulrahman Bin Faisal University (IRB-2017-01-206). This study utilized the data collected for stroke due to intracranial LAA under grant number 2017-199 Med.

### 2.1. Patients

Patients aged >18 years with ICLAD-related AIS were included in this study. The diagnosis of AIS was confirmed according to recommendations by the World Health Organization based on clinical information and neuroimaging (12). ICLAD was defined as ≥50% stenosis of the vessel lumen in the intracranial segment of the internal carotid artery, the M1/M2 segment of the middle cerebral artery, the A1 segment of the anterior cerebral artery for the anterior circulation, and the P1 segment of the posterior cerebral artery, vertebral artery, or basilar artery for the posterior circulation (13). The presence of ICLAD in the artery responsible for triggering the AIS as the etiology of the presenting event was confirmed using vascular imaging [computed tomography angiography (CTA), magnetic resonance angiography (MRA), or conventional cerebral angiogram].

The findings and diagnoses were verified by a stroke consultant and a neuroradiologist. Patients with diagnoses other than AIS, such as intracerebral hemorrhage, space-occupying lesions, cerebral venous sinus thrombosis, or central nervous system infections, were excluded. Patients with etiologies other than ICLAD for AIS [cardio-embolism (history of atrial fibrillation and intracardiac thrombus or other causes of embolic stroke from heart), small-vessel occlusion, other determined and undetermined etiologies, extracranial large artery disease], or an incomplete diagnostic workup were also excluded.

### 2.2. Data collection

**Figure 1:**
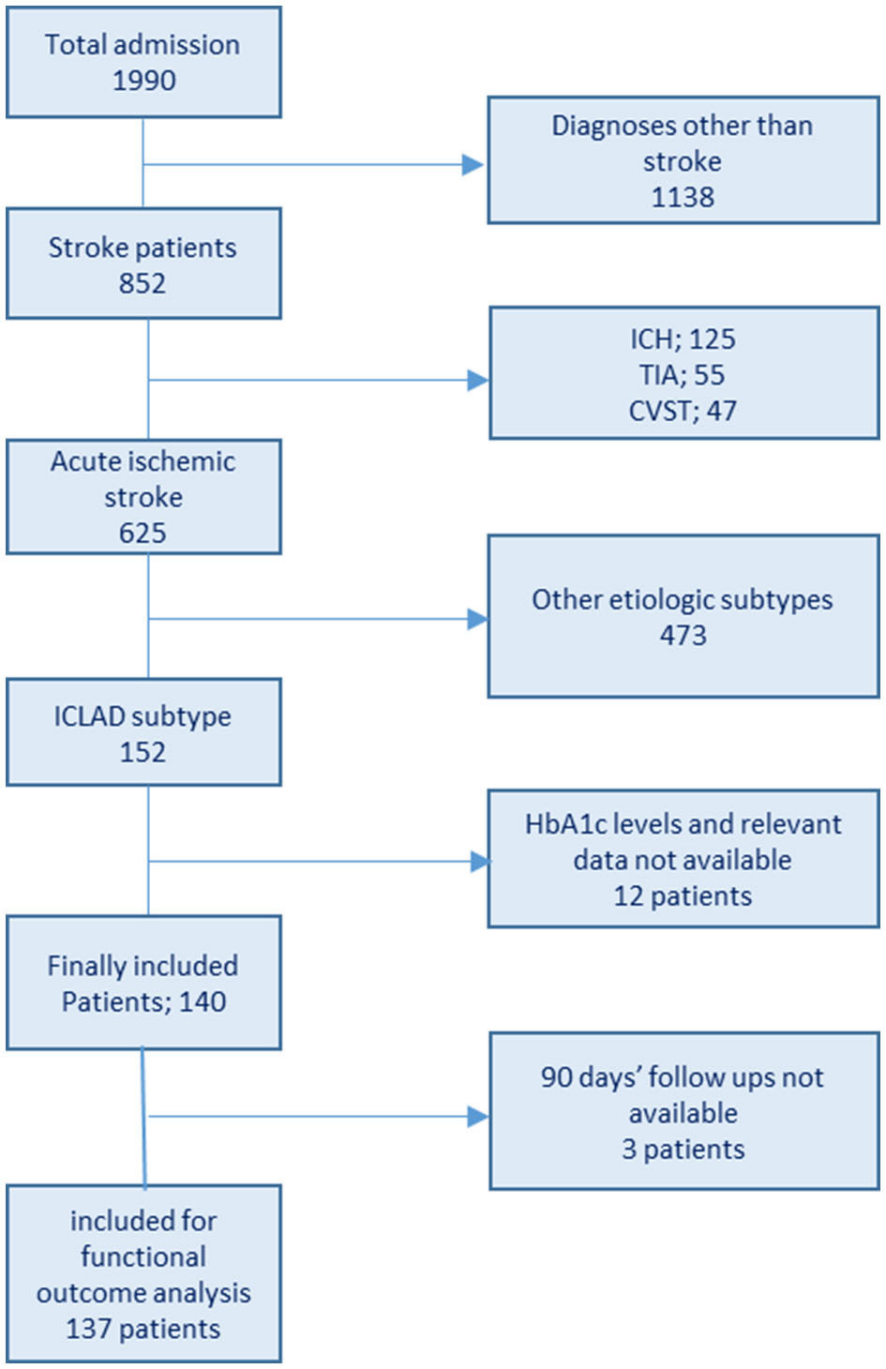
Flowchart of patient enrollment.

Data were collected by reviewing the electronic medical charts of patients from the hospital data bank. The data collection method and definitions were largely the same as described previously (2). The patient inclusion flowchart is shown in Figure 1. Baseline clinical information of all enrolled patients, including age, sex, admission National Institutes of Health Stroke Scale (NIHSS) score, history of DM, hypertension (HTN), dyslipidemia (DLP), prior stroke or transient ischemic attack (TIA), and smoking habits were collected from the hospital database. Laboratory findings included fasting blood sugar (FBS), admission blood sugar, HbA1c, and fasting lipid profile [total cholesterol (TC), triglycerides (TGs), low-density lipoprotein (LDL) cholesterol, and high-density lipoprotein (HDL) cholesterol]. Brain computed tomography and/or magnetic resonance imaging and CTA/MRA/cerebral angiogram data were also assessed. Infarct patterns were defined based on neuroimaging findings as follows: territorial, watershed/border zone, and perforator/lacunar. Underlying mechanisms were categorized as follows: artery-to-artery embolism, thrombotic occlusion, hemodynamic impairment/hypoperfusion, and perforator/penetrating branch occlusion in continuation of parent stenosed vessel, as mentioned previously (2).

Other radiological characteristics included the area of circulation affected, the presence of large vessel occlusion (LVO), and malignant transformation. FOC was assessed using a 90-day modified Rankin Scale (mRS) as favorable (scores: 0–2) or unfavorable (scores: 3–6) (14). Mortality during the hospital stay and in-hospital complications were also noted. In-hospital complications included hospital-acquired infections (HAIs), deep venous thrombosis, pulmonary embolism, and acute kidney injury (AKI). HAIs are defined as nosocomial infections that are acquired after hospitalization and manifest at least 48 h after arrival at the hospital. They are diagnosed based on clinical signs and symptoms along with laboratory evidence and mainly include nosocomial pneumonia, urinary tract infections, and intravenous line infections (15). Our hospital has an infectious disease team for diagnosing and treating HAIs. AKI was diagnosed by nephrologists and was defined as an increase in serum creatinine of least 0.3 mg/dl within 48 h or 1.5 times the baseline value within 1 week, or a decrease in urinary output to 0.5 ml/kg/h for at least 6 h (16). HbA1c levels were measured using high-performance liquid chromatography using a G8 HPLC analyzer^®^ (Tosoh Bioscience Inc., San Francisco, California, USA) according to the National Glycohemoglobin Standardization Program guidelines (17).

### 2.3. Statistical analysis

Data were analyzed using Statistical Package for the Social Sciences software, version 22.0 (IBM Corp., Armonk, New York, USA). The results are presented as frequencies and percentages for categorical variables, including sex, DM, HTN, smoking, DLP, and past history of stroke or TIA. The mean ± standard deviation (SD), median, and interquartile range (IQR) were used to describe quantitative variables. An independent *t*-test was used to compare means, and a chi-square test or Fisher's exact test was used to compare categorical variables. Receiver operating characteristic (ROC) curve analysis was used to determine the sensitivity and specificity of HbA1c for unfavorable FOC. The Youden index was used to determine the optimal cutoff for HbA1c. Patients were divided into two groups according to the optimal cutoff value. A two-tailed *p-*value of <0.05 was set as statistically significant. Differences in mortality and 90-day FOC between the two groups were evaluated using Pearson's test. The associations between in-hospital complications, mortality, and HbA1c were studied using univariate and multivariate analyses. Multivariate logistic regression analysis (MLR) was performed to adjust for variables that were found to be significantly associated with unfavorable outcomes in the univariate analysis (model 1) and atherosclerosis risk factors (model 2); statistical significance was set at a *p*-value of < 0.05. Analysis was performed only in patients with diabetes as well after excluding those without diabetes to find an association in the former group.

## 3. Results

In total, 152 patients diagnosed with ICLAD-related AIS were admitted during the study period. After excluding patients with no available data on HbA1c and relevant data, 140 were enrolled in the study (Figure 1). The median age of the patients was 59.5 (IQR: 49.8–72) years. Three-fourth of the patient population were men (male:female, 3:1). The median NIHSS score was 6.5 (IQR: 4–12), and the median HbA1c level was 7.4 (IQR: 6.2–9.9)%. The majority of the participants were patients with diabetes. HbA1c ≥7%, reflecting poor glycemic control, was found in 58.6% of all patients and 74.5% of the patients with diabetes.

**Figure 2:**
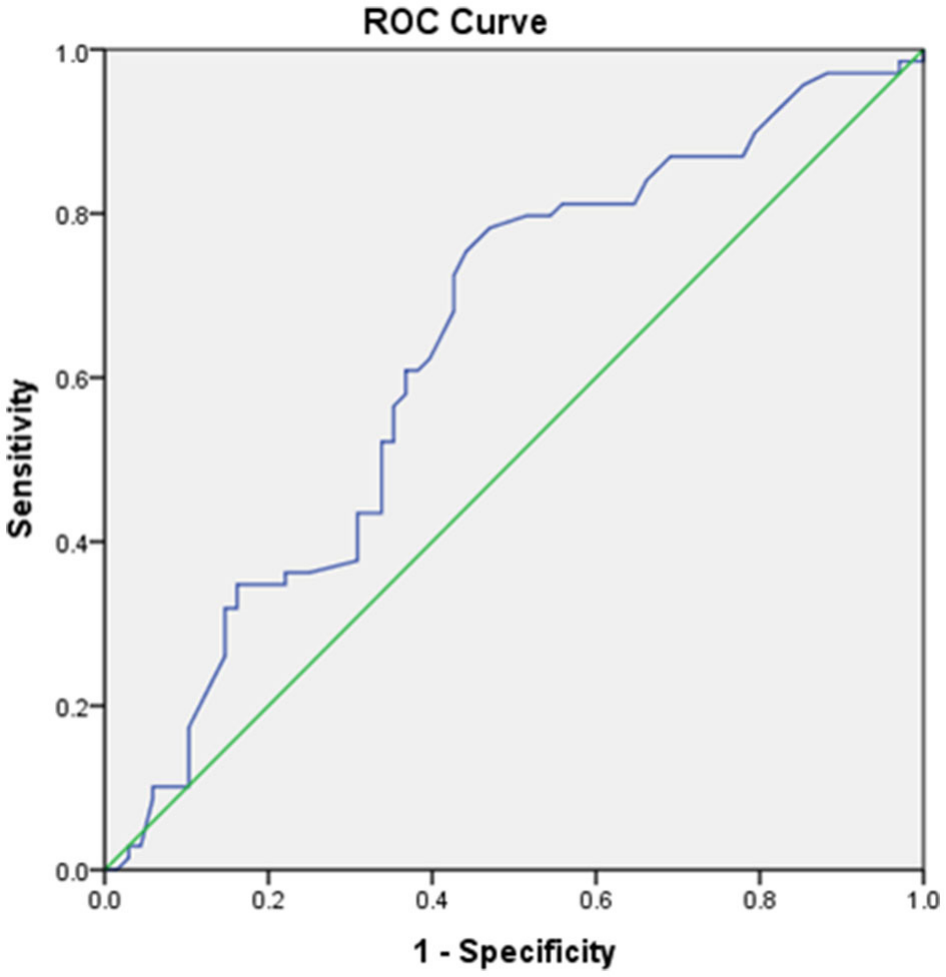
Receiving operating characteristic (ROC) curve showing the area under the curve (AUC) and sensitivity and specificity of the optimal cutoff glycated hemoglobin A1c (HbA1c) value for predicting unfavorable functional outcome.

ROC analysis was used to determine that the optimal predictive HbA1c value for unfavorable FOC was 6.95%. HbA1c was a good predictive factor, with an area under the ROC curve (Figure 2) of 0.639 [*p* = 0.005, 95% confidence interval (CI) = 0.55–0.73] and the largest Youden index of 0.313. The optimal cutoff was 6.95%, with a sensitivity of 75.4% but a relatively low specificity of 55.9%. The baseline demographic, clinical, and biochemical characteristics of all enrolled patients stratified according to the optimal cutoff of HbA1c level are summarized in Table 1.

**Table 1:** Demographic and clinical characteristics of patients and their comparison based on the HbA1c level.

**Characteristics**	**Total (*n* = 140)**	**HbA1c ≤6.9% (*n* = 58)**	**HbA1c > 6.9% (*n* = 82)**	***p*-value**	**Fisher's exact test**	**OR (95% CI) where applicable**
**Demographic**						
Age (mean ± SD)	59.95 ± 13.83	59.66 ± 15.68	60.18 ± 12.30	0.82	–	–
Sex; male	105 (75)	44 (75.9)	61 (74.4)	0.84	1.00	0.92 (0.42–2.01)
**Vascular risk factors**						
Diabetes mellitus, *n* (%)	110 (78.6)	28 (48.3)	82 (100)	<0.001	0.00	
Hypertension, *n* (%)	116 (82.9)	42 (72.4)	74 (90.2)	0.006	0.01	3.52 (1.39–8.92)
Coronary artery disease, *n* (%)	27 (19.3)	10 (17.2)	17 (20.7)	0.60	0.66	1.25 (0.52–2.98)
Dyslipidemia, *n* (%)	82 (58.6)	27 (46.6)	55 (67.1)	0.015	0.02	2.33 (1.17–4.67)
Smoker, *n* (%)	42 (30)	19 (32.8)	23 (28)	0.54	0.57	0.80 (0.38–1.66)
Previous stroke, *n* (%)	43 (30.7)	12 (20.7)	31 (37.8)	0.03	0.04	2.33 (1.07–5.06)
**Clinical and radiological variables**						
NIHSS score on presentation (mean ± SD)	7.92 ± 5.62	8.02 ± 5.29	7.84 ± 5.88	0.86		–
Arrival within the therapeutic window, *n* (%)	33 (23.6)	16 (26.6)	17 (22.8)	0.34	0.41	0.68 (0.31–1.51)
**Pattern of stroke**, ***n*** **(%)**						
Territorial	93 (66.4)	40 (69)	53 (64.6)	0.69	0.71	0.86 (0.42–1.78)
Border zone	26 (18.6)	12 (20.7)	14 (17)	0.77	0.82	0.88 (0.36–2.10)
Subcortical	17 (12.1)	04 (6.9)	13 (15.9)	0.11	0.12	2.54 (0.78–8.24)
Unidentified	04 (2.9)	02 (3.4)	02(2.4)	0.24	0.40	0.37 (0.06–2.09)
Anterior circulation stroke	93 (66.4)	41 (70.7)	52 (63.4)	0.36	0.46	0.71 (0.34–1.48)
Large vessel occlusion, *n* (%)	56 (40)	22 (37.9)	34 (41.5)	0.67	0.72	1.15 (0.58–2.30)
**Acute stroke-specific treatment**, ***n*** **(%)**						
Thrombolysis	21 (15)	12 (20.7)	09 (11)	0.12	0.15	0.48 (0.19–1.24)
Thrombectomy	12 (8.6)	05 (8.6)	07 (8.5)	0.98	1.00	0.98 (0.29–3.28)
**Biochemical values**						
Fasting blood glucose (mean ± SD)	159.75 ± 77.92	118.2 ± 40.79	190.29 ± 84.57	<0.001	–	–
HbA1c (mean ± SD)	8.31 ± 2.52	6.07 ± 0.84	9.90 ± 2.04	<0.001	–	–
Triglycerides (mean ± SD)	142.49 ± 123.73	116.34 ± 55.25	161.94 ± 153.89	0.03	–	–
Cholesterol/HDL ratio	4.55 ± 5.24	4.03 ± 4.95	4.72 ± 5.69	0.04	–	–
**Other variables**						
Death, *n* (%)	15 (10.7)	03 (5.2)	12 (14.6)	0.07	0.09	1.42 (1.06–1.92)
In-hospital complications, *n* (%)	30 (21.4)	08 (13.8)	22 (26.8)	0.06	0.09	1.34 (1.02–1.77)

**Figure 3:**
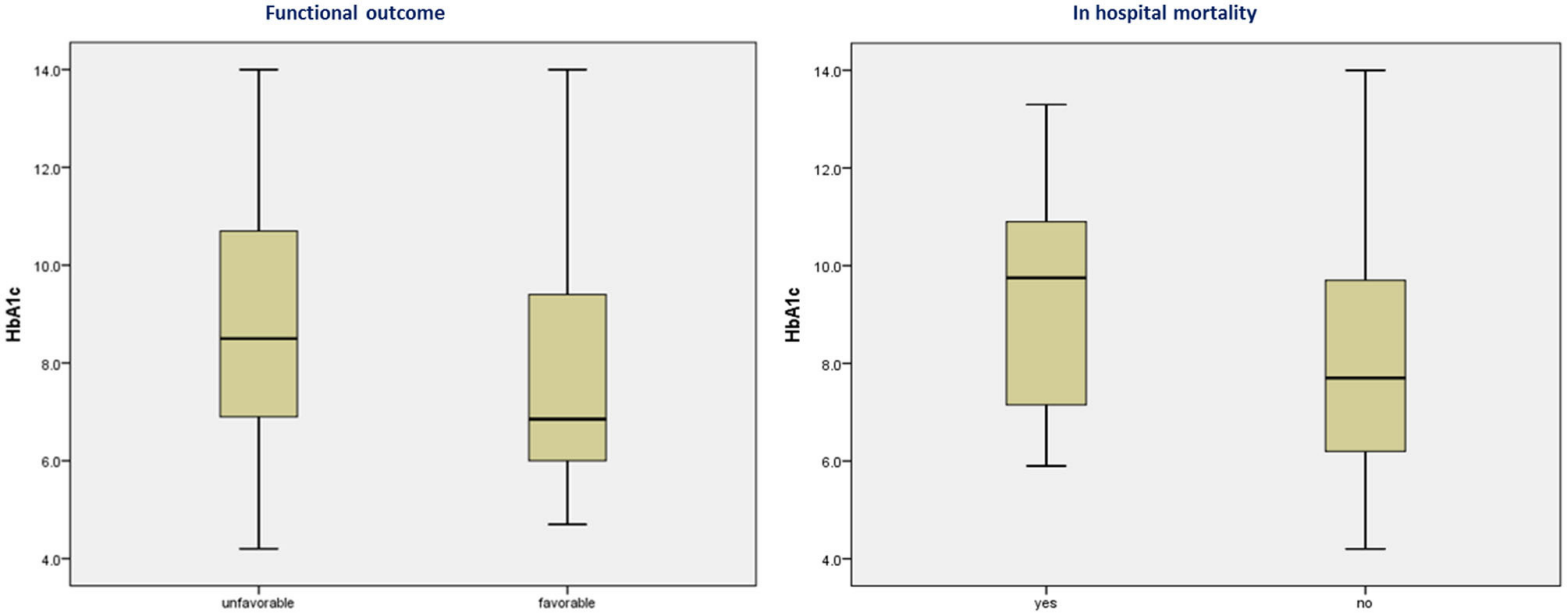
Box plot showing the distribution of glycated hemoglobin A1c according to functional outcomes and mortality.

The age and sex ratios were similar; however, the distribution of risk factors differed between the two groups. HTN, DLP, and history of stroke were significantly more frequent in the group with HbA1c >6.9% than in the group with HbA1c ≤6.9%. Stroke severity (*p* = 0.86) and NIHSS scores did not differ between the two groups. Compared to patients with HbA1c ≤6.9%, patients with HbA1c >6.9% had significantly higher serum TG levels (*p* = 0.03) and TC-to-HDL ratios (*p* = 0.04). However, the TC, LDL, and HDL levels did not differ between the two groups. There were no significant differences between the groups in the affected area of circulation, stroke pattern, presence of LVO, or malignant transformation. Moreover, the number of patients receiving stroke-specific treatments did not differ significantly between the two groups. Odds ratios (ORs) were higher for patients with HbA1c >6.9% who died but were not significant. However, the mean ± SD HbA1c differed significantly between patients who died (10.18 ± 2.56%) and those who were discharged (8.29 ± 2.50%) from the hospital (*p* = 0.002). The distribution of HbA1c according to FOC and mortality is shown as a box plot in Figure 3. The commonest cause of death was early neurological complications of fatal stroke, such as brain edema and herniation, leading to brain stem dysfunction among patients who died; two patients had internal carotid artery occlusion, five had complete middle cerebral artery occlusion, and three had posterior circulation infarction with brainstem involvement. Other causes of death were HAI with multiorgan dysfunction, pulmonary embolism, and acute coronary syndrome. HAIs (*n* = 25) were the most common in-hospital complications, followed by AKI (*n* = 4) and pulmonary embolism (*n* = 2). Sepsis with multiorgan dysfunction was the cause of AKI in two patients and multiple reasons in the other two patients. Three patients were lost to follow-up; therefore, the FOC was assessed in 137 patients. A comparison of different clinical and radiological parameters between patients with favorable and unfavorable FOC is presented in Table 2. FOCs differed significantly between the two groups (HbA1c >6.9% and HbA1c ≤6.9%), and there was a strong positive and significant correlation between HbA1c >6.9% and unfavorable FOC (*p* = <0.001). This association was also observed in patients with diabetes after excluding those without diabetes (*p* = 0.004). Admission blood sugar did not differ in patients who had unfavorable FOC than those who didn't. LVO is an important determinant of unfavorable FOC; therefore, this association was also assessed in patients without LVO and was found to be significant (*p* = <0.001).

**Table 2:** Demographic and other clinical variables in the favorable and unfavorable FOC groups.

**Variable**	**Favorable FOC (*n* = 68)**	**Unfavorable FOC (*n* = 69)**	**OR (95% CI)**	***p*-value**
Age (mean ± SD)	58.41 ± 15.14	61.12 ± 12.33	–	0.25
Male (103)	52 (50.5)	51 (49.5.6)	1.0 (0.68–1.48)	0.96
Diabetes mellitus (109)	48 (44)	61 (56)	1.95 (1.06–3.6)	0.01
Hypertension (114)	53 (46.5)	61 (53.5)	1.53 (0.85–2.76)	0.10
Coronary artery disease (26)	07 (27)	19 (73)	1.62 (1.18–2.21)	0.01
Prior H/O stroke (42)	16 (38.1)	26 (61.9)	1.36 (0.98–1.89)	0.07
Out of the therapeutic window (100)	50 (50)	50 (50)	1.03 (0.70–1.51)	0.88
NIHSS score on presentation (mean ± SD)	4.95 ± 3.91	10.84 ± 5.59	–	<0.001
Artery-to-artery embolism (67)	25 (37.3)	42 (62.7)	1.62 (1.14–2.30)	0.005
Large vessel occlusion (54)	19 (35.2)	35 (64.8)	1.58 (1.14–2.18)	0.006
Thrombolysis (21)	11 (52.4)	10 (47.6)	0.95 (0.58–1.54)	0.84
Fasting blood glucose (mean ± SD)	147.11 ± 64.03	176.25 ± 83.66		0.029
HbA1c (mean ± SD) 8.3 ± 2.4	7.80 ± 2.45	8.80 ± 2.4		0.018
Admission blood glucose (mean ± SD)	179 ± 91	208 ± 107		0.13
In-hospital complications (29)	06 (20.7)	23 (79.3)	1.86 (1.39–2.48)	<0.001
HbA1c ≤6.9% (55)	38 (69.1)	17 (30.9)	0.53 (0.37–0.74)	<0.001
HbA1c > 6.9% (82)	30 (36.6)	52 (63.4)	2.05 (1.33–3.14)	<0.001

Categorical data are shown as numbers (%).

Patients with unfavorable FOC did not differ in terms of age or sex; however, they had more severe stroke, higher NIHSS scores, and higher HbA1c than those with favorable FOC. The distribution of risk factors and underlying mechanisms of stroke also differed significantly between patients with unfavorable and favorable FOC. Data about antiplatelet and statin use were also collected. A significantly higher number of patients with HbA1c >6.9% were taking antiplatelets and statins; however, the prior use of antiplatelets and statins did not affect the FOC in our study cohort. Notably, thrombolysis and thrombectomy did not significantly affect the outcome in our cohort (Table 2). This could be attributed to the smaller number of patients receiving this treatment, thus preventing us from drawing reliable conclusions. In contrast, a higher number of patients receiving tissue plasminogen activator had favorable FOC rather than unfavorable FOC. Patients received intravenous thrombolysis within 3 h and underwent EVT within 6 h of symptoms onset, which was later extended to within 3–4.5 h and 24 h, respectively, according to the 2018 guidelines for AIS management (18). Standard thrombectomy devices (Merci retriever, Solitaire) ranging from 6 mm for proximal occlusion to 3 mm for distal occlusion, and standard aspiration catheters, either 5 F or 6 F, depending on the size of the target vessel, were used for thrombectomy.

**Figure 4:**
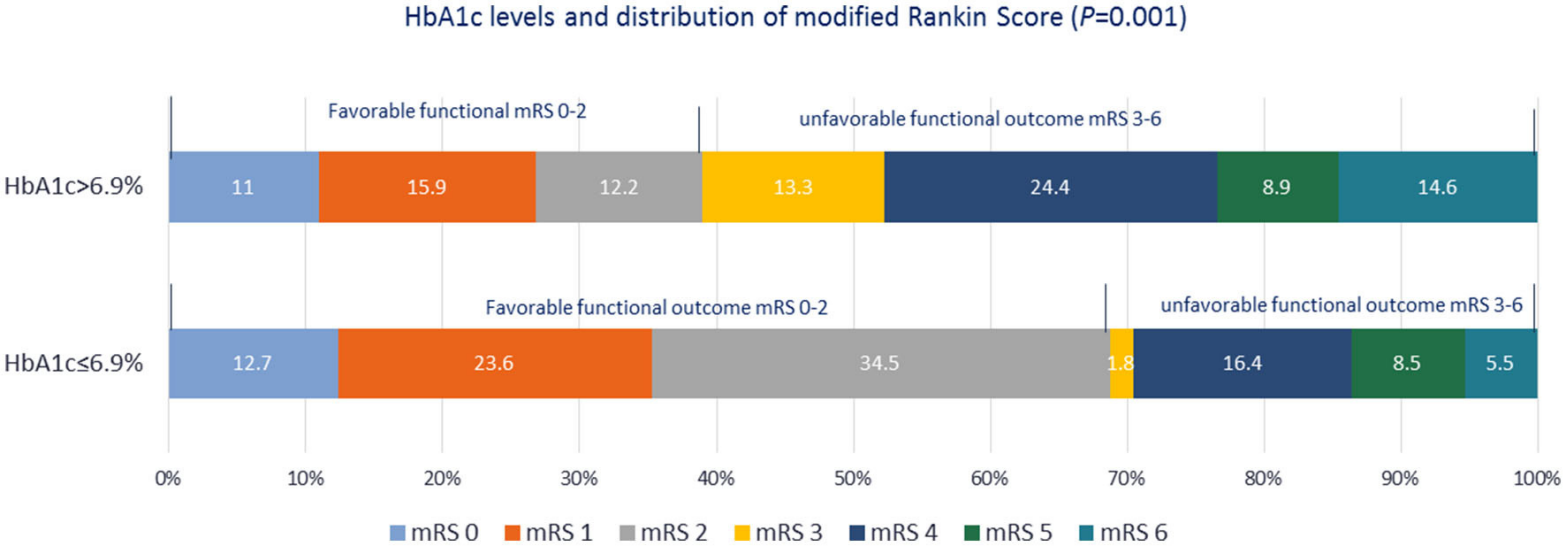
Distribution of modified Rankin Scale scores at the 3-month follow-up after stroke symptom onset in the two groups based on the glycated hemoglobin A1c level.

Among patients undergoing thrombectomy, only four achieved successful reperfusion (TICI score: 3) and two had partial recanalization, whereas two experienced intracerebral hemorrhage. Arriving at the hospital within the therapeutic window, thrombolysis and thrombectomy are important variables that affect the FOC in patients with AIS. When these variables were adjusted in the MLR, HbA1c >6.9% remained significantly associated with an unfavorable FOC. Importantly, the significant association of HbA1c >6.9% with unfavorable FOC was also sustained in the MLR analysis after adjusting for confounding factors, including DM, coronary artery disease (CAD), NIHSS score, infarct mechanism, LVO, and FBS, as detailed in Table 3. The wide 95% CI range observed can be explained by the small sample size. Patients with HbA1c ≤6.9% were less likely to have an unfavorable FOC. Although in-hospital complications and mortality rates were lower in patients with HbA1c ≤6.9% than in those with HbA1c >6.9%, a significant *p*-value was not achieved. The distribution of the mRS scores at 3 months between the two groups is shown in Figure 4. Another observation considering the coronavirus disease 2019 (COVID-19) pandemic was that HbA1c (8.73 ± 2.6 vs. 8.11 ± 2.4, *p* = 0.83) and FOC (*p* = 0.16) did not differ significantly between the COVID-19 and non-COVID-19 years.

**Table 3:** Significant association of unfavorable FOC with HbA1c >6.9% described by ORs and logistic regression.

**Patient group**	**HbA1c**>**6.9% and unfavorable FOC**		
	***p*** **-value**	**OR**	**95% CI**
**Univariate analysis**			
All patients	<0.001	2.05	1.33–3.14
Patients with diabetes only	0.004	1.67	1.25–2.24
Patients without large vessel occlusion	<0.001	3.40	1.58–7.32
**Multivariate analysis for all patients**			
Model 1^†^	0.008	6.89	1.64–28.8
Model 2^††^	0.01	3.46	1.33–9.01

† Adjusted for all confounders found to have a significant association with unfavorable FOC in univariate analysis (diabetes mellitus, coronary artery disease, large vessel occlusion, NIHSS score, artery-to-artery mechanism, fasting blood glucose level, and in-hospital complications).

†† Adjusted for atherosclerosis risk factors (diabetes mellitus, coronary artery disease, hypertension, dyslipidemia, and smoking).

We also assessed the associations between FOC and HbA1c <5.7 and <6.5% to highlight the association of the optimal cutoff HbA1c value with FOC. HbA1c <5.7% was associated with a lower likelihood of unfavorable FOC (*p* = 0.03, OR = 0.43, 95% CI = 0.15–1.18) in univariate analysis but not in MLR (*p* = 0.95, OR = 0.91, 95% CI = 0.04–20.7). HbA1c <6.5% did not show a significant association with FOC (*p* = 0.12).

## 4. Discussion

The present study demonstrated that HbA1c >6.9% was independently associated with an unfavorable FOC in patients with ICLAD-related stroke. The likelihood of a favorable FOC was significantly lower in patients with HbA1c >6.9% than in those with HbA1c ≤6.9%. Furthermore, mortality and in-hospital complications were more frequent in patients with HbA1c >6.9% than in those with HbA1c ≤6.9%. This correlation between HbA1c >6.9% and unfavorable FOC persisted even after adjusting for confounding and atherosclerosis risk factors. These results are similar to previously reported observations (8–10, 19, 20). However, none of these studies have separately described the effects of HbA1c on ICLAD-related stroke. Nevertheless, the association between poor FOC and HbA1c >8% as compared to HbA1c ≤6.5% and 6.5 to ≤8% in patients with the LAA stroke subtype compared to those with the non-LAA type has been recently established by Dong et al. (9). Similarly, Diprose et al. reported that HbA1c is an independent predictor of poor outcomes following EVT. They observed that increased HbA1c levels (per 10 mmol/mol) were significantly associated with poor FOC and higher mortality rates (21). In the present study, 56 (40%) patients had LVO, and only 12 (21.4%) of them underwent thrombectomy. There were different reasons for the other patients not undergoing thrombectomy, such as being out of the therapeutic window for intervention, having an established infarction, and treatment refusal by the family. As only a few patients underwent thrombectomy and the rate of successful reperfusion was not high, the beneficial effect of thrombectomy could not be observed in our study. Hence, no meaningful conclusions can be drawn to second the observation made by Diprose et al. (21). Nevertheless, this valuable observation requires further investigation in prospective studies as a substantial number of patients with ICLAD present with LVO and needs thrombectomy in an acute setting; therefore, correlating the outcome with HbA1c levels in these patients could have important therapeutic implications.

In an 8-year follow-up study, HbA1c ≥8.5% was significantly associated with an increased risk of CVD (hazard ratio = 2.11; 95% CI = 1.37–3.25) and stroke mortality (hazard ratio = 2.43; 95% CI = 1.06–5.55) compared to HbA1c levels of 7.5%−8.4% (22). ORs for mortality were higher among patients with HbA1c >6.9% compared to those with HbA1c ≤6.9% in our study as well. Although we did not investigate the stroke recurrence rate, higher rates of stroke recurrence in the Chinese population have been previously reported (20). Notably, the association of HbA1c with poor FOC and mortality in patients with AIS has been proven in both patients with and without diabetes as studies have demonstrated that the adjusted ORs for unfavorable FOC and mortality increased significantly with increasing HbA1c terciles, both among patients with and without diabetes. Furthermore, these relationships were independent of other confounding factors (21, 23). However, in contrast to these studies, another study found that HbA1c was an independent predictor of unfavorable FOC only in patients with diabetes with AIS, with the LAA etiologic subtype (8). These conflicting data require further investigation to confirm the actual association between HbA1c and AIS outcomes in both patients with and without diabetes. We investigated only patients with diabetes and all patients separately, and the effect of HbA1c >6.9% on FOC was significant in both groups.

Regarding the risk-factor distribution, HTN, DLP, and history of stroke were statistically more prevalent, and ORs were higher for CAD in the group of patients with HbA1c >6.9% as compared to patients with HbA1c ≤6.9%. Another important observation was that the mean TG level and cholesterol/HDL ratio were significantly higher in patients with HbA1c >6.9% than in those with HbA1c ≤6.9%. Hypertriglyceridemia is an important modifiable risk factor for atherosclerotic pathology and has been reported to be significantly associated with intracranial artery stenosis (24). Higher TG levels in patients with diabetes emphasize the need for proper management of DLP in these patients.

Several theories have been proposed to explain the relationship between chronic hyperglycemia, vascular atherosclerosis, and injury. These include endothelial injury (25) and morning blood pressure surges, which accelerate vascular damage by promoting inflammation in atherosclerotic plaques (26, 27) as well as the recently confirmed hyperglycemic memory theory (28). The hyperglycemic memory theory suggests that hyperglycemia induces trained immunity, promoting the transformation of macrophages from the anti-inflammatory to the pro-inflammatory type, which further aggravates atherosclerotic lesions (29). It is important to assess chronic hyperglycemia in patients at high risk of cerebral atherosclerosis. HbA1c, as compared to FBS, reflects the average glycemic control over the past 120 days, which reduces the potential for misdiagnosis caused by stress hyperglycemia (30). The ADA has defined HbA1c ≥7% as indicative of inadequate glycemic control and has recommended diet, exercise, and hypoglycemic drugs, including insulin, to achieve glycemic control (11). In our cohort, more than half of the total patients and approximately three-fourth of the patient population with diabetes had HbA1c ≥7%, suggesting that a large number of patients did not meet the recommendation of the ADA, which is an alarming observation. It is important to make healthcare practitioners aware of this observation, as strict management to lower HbA1c below 7%, which is comparable to the predictive HbA1c value ( ≤6.9%) identified in our study, can improve the outcome of ICLAD-related stroke and prevent functional disabilities. Importantly, ICLAD-related stroke has a poor prognosis; therefore, the need to control independent modifiable predictors of unfavorable outcomes should not be overlooked (31). The International Diabetic Federation has reported that SA ranks fourth among the top five countries for the number of people with diabetes among the Middle East and North African countries and predicted that approximately one-quarter of Saudi adults will have diabetes by 2045 (32). The prevalence of diabetes in Saudi adults is 17.7% (33), and alarmingly, a 10-fold increase in diabetes cases has been observed in the past few decades, making it a significant health burden in SA (34, 35). It is, therefore, crucial to optimize diabetes care in SA to minimize complications and reduce the disability resulting from uncontrolled diabetes. The Saudi National Diabetes Center, Saudi Health Council, launched comprehensive clinical practice guidelines in August 2021, which recommend that most patients with diabetes should achieve HbA1c <7% (36). Adherence to these guidelines by physicians and patients with diabetes can prevent the complications of DM.

Our study had certain limitations. First, it was a single-center study with a relatively small sample size, and fewer patients underwent thrombectomy and received intravenous thrombolysis; therefore, the results and observations are representative of a small, selected population, which limits our ability to draw meaningful conclusions. Second, this was a retrospective study, comprising mostly patients with diabetes, and some patients may have been missed because of missing or inadequate data, which could have impacted the study outcomes. However, some previous studies reported data with even smaller sample sizes than ours (10). Furthermore, we studied patients with a specific etiological subtype only rather than including all AIS patients. Third, the actual number of patients with ICLAD could be different as some patients were excluded because of an incomplete diagnostic workup or the non-availability of relevant information. Finally, the effect of anti-diabetic medications on FOC was not evaluated due to deficient data about medication use and adherence prior to stroke, and secondary outcomes, such as the incidence of recurrent stroke and 1-year mortality, which are important prognostic parameters in patients with AIS, were not addressed. Determining their association with HbA1c levels could have a great impact on management strategies. The strengths of the study are that we studied the association between FOC and predictive HbA1c value determined using ROC curve analysis in patients with ICLAD-related AIS, which has not been widely studied previously, and adjusted for all confounding factors, including DM, to prove the significance of the associations. We found a significant association of HbA1c >6.9% (comparable to ≥7%, defined as reflecting a lack of adequate glycemic control by the ADA) with FOC, which has important implications. Future larger-scale studies are required to evaluate the real relationships between HbA1c and ICLAD-related AIS outcomes and confirm our findings. Such studies can have higher statistical significance and reliability to be implemented in real clinical practice. The limitations in our study also need to be overcome by further studies. Meanwhile, the present study can be the initial step in the journey toward improved management of patients with stroke.

In the current study, HbA1c >6.9% was found to be significantly associated with unfavorable FOC in patients with ICLAD-related AIS, the subtype, which is highly prevalent and has not previously been studied solely for this association. Patients with HbA1c >6.9% were more likely to have an unfavorable FOC than those with HbA1c ≤6.9%. The magic HbA1c number identified in our study is different from that in previous studies and equivalent to ADA recommendations for adequate glycemic control, which makes it unique in supporting clinical practice guidelines. Approximately, three-fourth of our patient population with diabetes had HbA1c levels higher than that recommended by the ADA, which is worrisome. The findings of the present study have important prognostic and therapeutic implications and emphasize the need to implement proper glycemic control programs to reduce unfavorable outcomes in patients with stroke. There is an unmet need for further large sample-size prospective studies investigating the relationship between HbA1c and AIS, including its effect on outcomes.

## Data availability statement

The raw data supporting the conclusions of this article will be made available by the authors, without undue reservation.

## Author contributions

AZ drafted the manuscript and was responsible for the conception and design of this study. AAlb, RS, and SN collected and assessed the data, analyzed the results, and contributed to the writing of the results. MZ contributed to data analysis and results writing. FA, DA, ES, and MA contributed to writing the discussion. HA-J, AAlA, AAlS, AAld, and FJGA reviewed the manuscript and contributed to manuscript writing and revision. All authors contributed to the article and approved the submitted version.
